# Physical Properties and Shifting of the Extracellular Membrane Vesicles Attached to Living Bacterial Cell Surfaces

**DOI:** 10.1128/spectrum.02165-22

**Published:** 2022-11-16

**Authors:** Yousuke Kikuchi, Masanori Toyofuku, Yuki Ichinaka, Tatsunori Kiyokawa, Nozomu Obana, Nobuhiko Nomura, Azuma Taoka

**Affiliations:** a Institute of Science and Engineering, Kanazawa University, Kakuma, Kanazawa, Japan; b WPI Nano Life Science Institute (WPI-NanoLSI), Kanazawa University, Kakuma, Kanazawa, Japan; c Faculty of Life and Environmental Sciences, University of Tsukuba, Tennodai, Tsukuba, Japan; d Microbiology Research Center for Sustainability (MiCS), University of Tsukuba, Tennodai, Tsukuba, Japan; e Suntory Rising Stars Encouragement Program in Life Sciences (SunRiSE), Seika, Kyoto, Japan; f Graduate of Life and Environmental Sciences, University of Tsukuba, Tennodai, Tsukuba, Japan; g Transborder Medical Research Center, Faculty of Medicine, University of Tsukuba, Tennodai, Tsukuba, Japan; University of Guelph

**Keywords:** extracellular membrane vesicles, cell-cell communication, quorum sensing, cell surface, live-cell imaging, atomic force microscopy, physical properties mapping

## Abstract

Bacterial cells release nanometer-sized extracellular membrane vesicles (MVs) to deliver cargo molecules for use in mediating various biological processes. However, the detailed processes of transporting these cargos from MVs to recipient cells remain unclear because of the lack of imaging techniques to image nanometer-sized fragile vesicles in a living bacterial cell surface. Herein, we quantitatively demonstrated that the direct binding of MV to the cell surface significantly promotes hydrophobic quorum-sensing signal (C16-HSL) transportation to the recipient cells. Moreover, we analyzed the MV-binding process in the Paracoccus denitrificans cell surface using high-speed atomic force microscopy phase imaging. Although MV shapes were unaltered after binding to the cell surface, the physical properties of a group of single MV particles were shifted. Additionally, the phase shift values of MVs were higher than that of the cell’s surfaces upon binding, whereas the phase shift values of the group of MVs were decreased during observation. The shifting physical properties occurred irreversibly only once for each MV during the observations. The decreasing phase shift values indicated alterations of chemical components in the MVs as well, thereby suggesting the dynamic process in which single MV particles deliver their hydrophobic cargo into the recipient cell.

**IMPORTANCE** Compared to the increasing knowledge about MV release mechanisms from donor cells, the mechanism by which recipient cells receive cargo from MVs remains unknown. Herein, we have successfully imaged single MV-binding processes in living bacterial cell surfaces. Accordingly, we confirmed the shift in the MV hydrophobic properties after landing on the cell surface. Our results showed the detailed states and the attaching process of a single MV into the cell surface and can aid the development of a new model for MV reception into Gram-negative bacterial cell surfaces. The insight provided by this study is significant for understanding MV-mediated cell-cell communication mechanisms. Moreover, the AFM technique presented for nanometer-scaled mapping of dynamic physical properties alteration on a living cell could be applied for the analyses of various biological phenomena occurring on the cell surface, and it gives us a new view into the understanding of the phenotypes of the bacterial cell surface.

## INTRODUCTION

Bacteria use membrane vesicles (MVs) as delivery devices for extracellular transportations of materials, such as genetic materials for horizontal gene transfer, toxins for killing their competitors, and signaling molecules for cell-cell communication (1–5). Therefore, there has been extensive research on this topic in not only bacteriology fields but also applied microbiology, medicine, bioengineering, plant science, and agriculture fields (6–9). Recent information from cutting-edge imaging techniques has also expanded our knowledge regarding the detailed mechanisms of MV biogenesis processes (10). MVs are produced via several mechanisms, *viz.*, outer membrane blebbing (11–14), explosive cell lysis (15), and bubbling cell death (16). Nevertheless, compared with the increasing knowledge about how MVs launch from donor cells, our knowledge about how recipient cells receive cargo from MVs remains limited.

MV binding and subsequent fusion to the recipient cells are also thought to be essential steps for receiving the cargo. Similarly, Kadurugamuwa and Beveridge observed the binding of Pseudomonas aeruginosa (17) and Shigella flexneri (18) MVs to other bacterial cell surfaces using transmission electron microscopy. They showed possible fusion sites of these MVs to the outer membranes. Additionally, using fluorescence microscopy, several reports demonstrated attachments of fluorescence-labeled MVs to cells receiving cargo molecules from MVs. Toyofuku et al. (19) also showed the process of receiving quorum-sensing (QS) signal molecules in fluorescence-labeled MVs during the binding of cells. Moreover, Qiao et al. (20) investigated MV-mediated plasmid DNA transfer to Escherichia coli cells through fluorescence-labeled MVs. Fluorescence imaging was also used in binding assays involving MVs on cell surfaces (19, 21–23). Furthermore, Bos et al. (24) achieved visualization and tracking behavior of large-sized MVs (e.g., >200 nm in diameter) in live bacteria using fluorescence microscopy and showed that MVs moved in proximity to the bacterial surface. Although understanding the detailed MV-receiving process by a recipient cell is needed for the comprehensive characterization of MV-mediated transportation processes, previous imaging experiments had limitations related to spatial and temporal resolutions or invasiveness of specimens. These limitations prevented the unveiling of details about the MV-receiving processes on a living cell’s surface.

Hence, as a complementary technique in this study, we used the high-speed atomic force microscopy (AFM) technique for visualizing nanometer-sized vesicle structures and studied their dynamic behavior in a living cell’s surface in the liquid. High-speed AFM enabled studying the imaged structural dynamics of biological specimens with nanometer spatial and subsecond temporal resolutions (25–27). Additionally, although high-speed AFM is mainly used for conducting *in vitro* observations of working protein molecules, such as myosin V (28), F_1_-ATPase (29), and CRISPR-Cas9 (30), using purified proteins (26), we used high-speed AFM to perform *in vivo* imaging of living bacterial cell surface structures (31, 32). Furthermore, phase imaging is among the AFM imaging modes used for mapping the physical properties of a sample’s surface (33–35). Previously as well, we used high-speed AFM phase imaging for a single MV particle analysis and revealed that bacterial cells generated physically heterogeneous types of MVs (36).

Therefore, in this study, to elucidate what happens to a single MV particle after landing on a cell’s surface, we employed both high-speed AFM imaging modes, topographic, and phase-imaging techniques to simultaneously obtain structural information and physical states of MVs in living bacterial cell surfaces. Accordingly, we imaged MV-binding processes and confirmed the shifting hydrophobic properties of MVs, which are proposed to be attributed to the chemical composition of MVs, thereby suggesting the diffusion of MV contents into the cell surface. Our findings provide a model for the transportation of MVs into Gram-negative bacterial cell surfaces.

## RESULTS

### MVs mediate QS-signal delivery.

To assess the MV-receiving procedure in recipient cells, we focused on its mediated QS-signal transportation processes between bacterial cells. Lipid bilayer membranes consisting of MVs were expected to be powerful carriers for a group of hydrophobic QS-signal molecules (19, 22, 37), because these molecules were thought to be difficult to transmit via diffusion in the extracellular milieu between two cells. Notably, MV-mediated delivery is expected to have advantages, including species-specific transportation and the ability to send signals to cells at distant locations and preserve signal molecules for a long time at particular locations, for example, in biofilm (19, 37).

**FIG 1:**
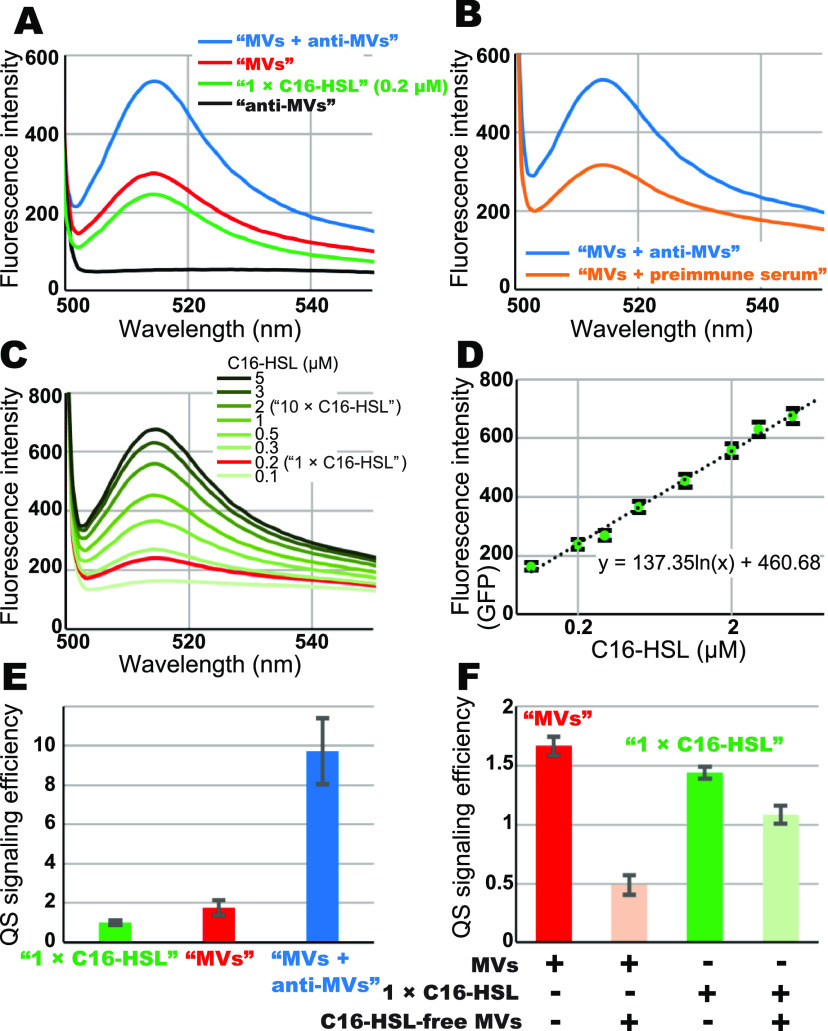
Efficiencies of MV-mediated QS-signal transportation. (A) Fluorescence spectra obtained from P. denitrificans AHL reporter cell suspensions incubated with MVs; 1.1 × 10^9^ particles/mL (“MVs” treatment, red), 0.2 μM C16-HSL (“1× C16-HSL” treatment, green), MVs plus anti-MV antibodies (“MVs + anti-MVs” treatment, blue), and anti-MV antibodies only (“anti-MVs” treatment, black). (B) Fluorescence spectra of AHL reporter strain cell suspensions of “MVs + anti-MVs” treatment (blue), and MVs plus preimmune serum (“MVs + preimmune serum” treatment, orange). (C) Fluorescence titration spectra of the AHL reporter strain cell suspensions incubated with PBS containing C16-HSL ranging between 0.1 and 5 μM. (D) Relationship between C16-HSL concentrations and GFP’s fluorescence intensities from AHL reporter cell suspensions. The values of GFP fluorescence intensities observed were from the emissions at 515 nm from panel C. Green circles, error bars, and a dashed line indicate experimental values, standard errors, and a fitted curve, respectively. The equation of the fitted curve is shown. (E) QS-signaling efficiencies were exhibited based on corresponding C16-HSL concentrations required to express the same GFP fluorescence intensity in the cell’s suspension, using the equation in panel D. The GFP’s fluorescence intensity from the “1× C16-HSL” treatment was defined as one. “1× C16-HSL,” “MVs,” and “MVs + anti-MVs” treatments are shown as green, red, and blue bars, respectively. Data are presented as means ± SE (bars) (*n* = 3). (F) The effect of C16-HSL-free MVs on MVs mediated signal transportation. C16-HSL-free MVs (1.1 × 10^10^ particles/mL) were added in “MVs” and “1× C16-HSL” treatments. QS-signal efficiencies were estimated from the cell suspensions incubated with MVs (1.1 × 10^9^ particles/mL; “MVs” treatment, red), “MVs” treatment plus C16-HSL-free MVs (1.1 × 10^10^ particles/mL, light red), 0.2 μM C16-HSL (“1× C16-HSL” treatment, green), and “C16-HSL” treatment plus C16-HSL-free MVs (1.1 × 10^10^ particles/mL, light green). Data are presented as means ± SE (bars) (*n* = 3).

Hence, we explored the MV-receiving process through live-cell imaging using AFM and the Paracoccus denitrificans AHL reporter strain (19) as a specimen. This strain lacks AHL biosynthesis genes but has a *gfp* gene under a las promoter, which is activated by exogenous *N*-hexadecanoyl-l-homoserine lactone (C16-HSL) (38). First, we compared the efficiencies of QS-signal transportation processes by MV treatment and simple diffusion to quantitatively confirm the superiority of the MV-mediated transportation process in QS-based cell-cell interactions. C16-HSL-induced green fluorescent protein (GFP) intensities in AHL reporter cell suspensions were measured using a fluorometer (Fig. 1). The cells harvested from logarithmic growth cultures were then incubated for 3 h with phosphate-buffered saline (PBS) containing 0.2 μM C16-HSL (termed as the “1× C16-HSL” treatment) or PBS containing MVs isolated from wild-type P. denitrificans (1.1 × 10^9^ particles/mL) (“MVs” treatment). The solution now containing 1.1 × 10^9^ particles/mL P. denitrificans MV solution also contained 0.2 μM C16-HSL according to the mass spectrometry measurements performed as reported previously (19). Figure 1A shows the GFP emission fluorescence spectra obtained on evaluating the cell suspensions. GFP fluorescence intensities of cells treated with “MVs” were 1.25 times higher than that of “1× C16-HSL”-treated cells. We prepared polyclonal antibodies against P. denitrificans MVs by surgically implanting purified and suspended MVs as antigens in a rabbit. The anti-MV antibodies obtained recognized both of MVs (Fig. S1A to D in the supplemental material) and the cell surface of P. denitrificans (Fig. S1E and F) as confirmed via immunogold and immunofluorescence stainings, respectively. Furthermore, the AHL reporter cells incubated with PBS containing MV and anti-MV antibodies, 13 μg IgG proteins/mL (“MVs + anti-MVs” treatment), showed a GFP fluorescence intensity 2.24 times higher than that of “1× C16-HSL”-treated cells (Fig. 1A). Treatment with anti-MVs antibody alone without MVs (“anti-MVs” treatment) induced no GFP signals, indicating that the anti-MV antibody itself had no effect on the GFP expression in the AHL reporter cells (Fig. 1A). Additionally, the AHL reporter cells incubated with PBS containing MVs and preimmune serum showed similar GFP fluorescence intensities with “MVs”-treated cells (Fig. 1B), indicating that enhancing the GFP expression is a specific phenomenon for anti-MV antibodies. These results suggest that the anti-MV antibodies enhance signal transportation from the MVs to the cells; however, the mechanism is unclear.

Subsequently, we titrated C16-HSL concentration and GFP fluorescence intensity (Fig. 1C). The C16-HSL concentration and GFP fluorescence intensity present a clear logarithmic relationship (Fig. 1D). Using this curve, we exhibited QS-signaling efficiencies. Figure 1E shows the QS-signaling efficiency of each treatment. Note that each “1× C16-HSL,” “MVs,” and “MVs + anti-MVs” treatment contained the same net amount of C16-HSL (approximately 0.2 μM). Results showed that the GFP’s fluorescence intensity from “MVs”-treated cells was equivalent to 0.34 μM that of C16-HSL-treated cells, indicating that MV-mediated C16-HSL transportation achieved a 1.7 times higher efficiency than the simple diffusion of 0.2 μM C16-HSL shown as “1× C16-HSL” (Fig. 1E). To confirm that the GFP signals of the “MVs”-treated cells were attributed to the C16-HSL signals contained in the MVs, we added C16-HSL-free MVs (1.1 × 10^10^ particles/mL; 10 times higher the concentration of C16-HSL-containing MVs) to the “MVs” or “1× C16-HSL” treatments (Fig. 1F). MVs prepared from the spent cultures of ALH reporter cells (Δ*pdn*I; LuxI-type AHL synthase mutant) were used as C16-HSL-free MVs. The QS signal efficiencies of “MVs” treatment were diminished by 70% when C16-HSL-free MVs were added (Fig. 1F and Fig. S2A). Although the QS signal efficiencies of the “1× C16-HSL” treatment were decreased by 25%, this might involve the adsorption of solute C16-HSL to the abundant MV membranes. These results showed that C16-HSL-free MVs competitively prevent MV-mediated C16-HSL transportation, indicating that GFP expression of the “MVs”-treated cells is attributed to C16-HSL signals contained in the MVs.

Additionally, the QS-signaling efficiency of the cell suspensions treated with “MVs + anti-MVs” was found to be 9.7 times higher than that of those treated with “1× C16-HSL” based on C16-HSL titrations (Fig. 1E). Hence, we tested three possible explanations for the signal transduction enhancement mechanism by the anti-MV antibodies. The first possibility is that anti-MV antibodies can facilitate soluble C16-HSL uptake into P. denitrificans cells from the extracellular solution. To confirm this possibility, we tested the influence of anti-MV antibodies on C16-HSL-dependent GFP expression efficiency using C16-HSL titration in AHL reporter cell suspensions (Fig. S2B and C). The angle of the titration curve slightly increased upon the addition of anti-MV antibodies (Fig. S2C). Although GFP fluorescence intensities induced by 0.2 μM C16-HSL were increased 1.18 times by the addition of the anti-MV antibodies, the QS-signaling efficiency in the presence of MVs could not be attributed to this slight increase. Thus, the anti-MV antibodies binding unlikely influences the uptake of soluble C16-HSL signal. The second possibility would be that anti-MV antibodies could disrupt MV structures and help to release C16-HSL from the MVs into the extracellular solution. After the MVs were incubated with anti-MV antibodies, the IgG-MV complex was removed using a protein-A Sepharose column to assess this possibility. The obtained flowthrough (IgG removed sample) was incubated with the AHL reporter cells (Fig. S2D). The resulting GFP fluorescence intensity was reduced by 53% compared to that of the “MVs + anti-MVs” treatment (Fig. S2D). Therefore, this result implied that anti-MV antibodies had not been associated with the release of C16-HSL from the MVs into the solution and also suggested that the IgG-MV complex itself would be required for enhancement. The third possibility is that anti-MV antibodies can promote MV adsorption onto the cell surfaces and enhance MV-mediated signal transportation. Further, we investigated the third possibility by analyzing correlations between adhering MV concentrations and GFP fluorescence intensities at the single-cell level.

### Correlations between adhering MVs and the QS-dependent GFP expression as estimated by live-cell fluorescence imaging.

**FIG 2:**
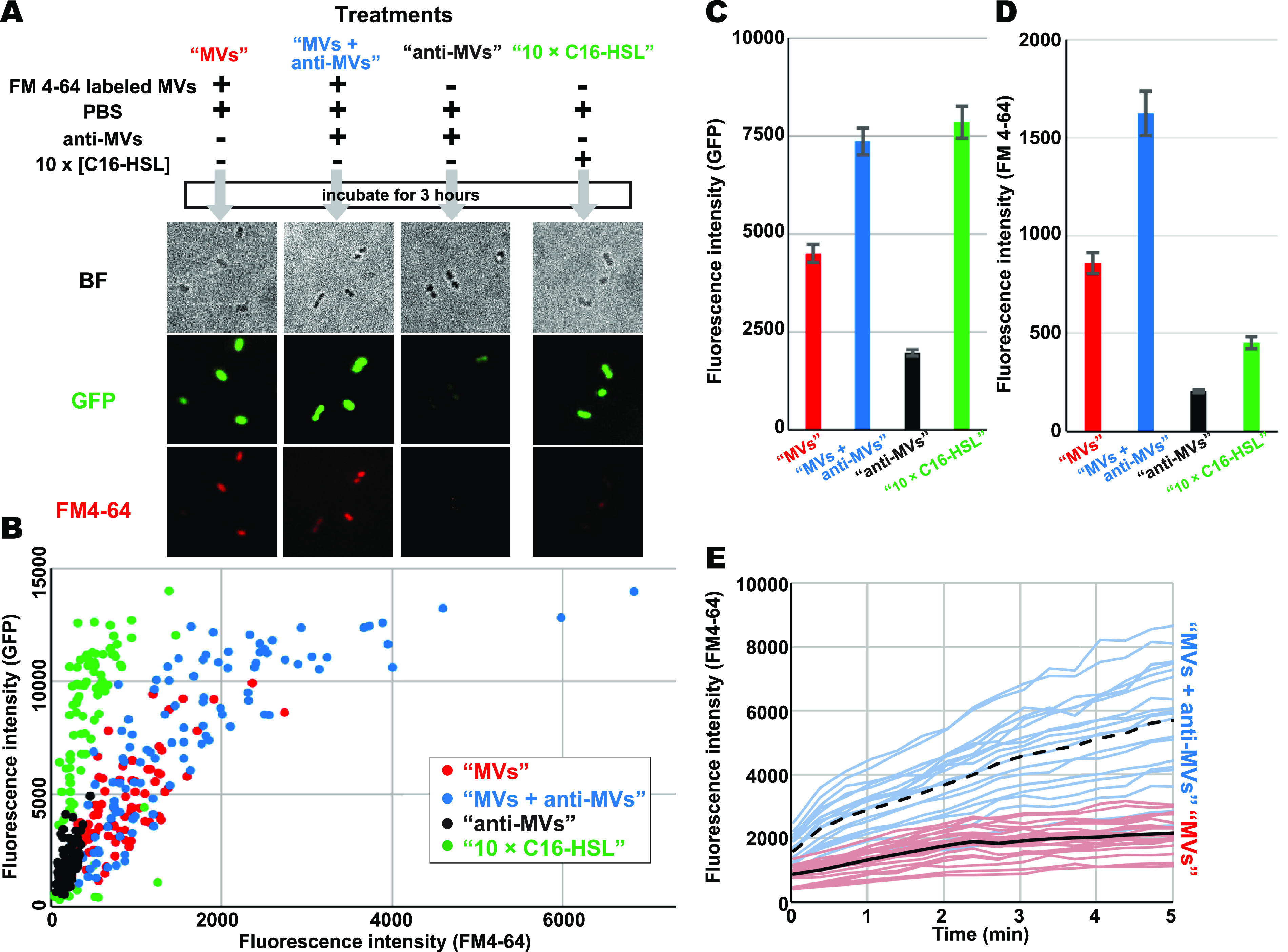
(A) AHL reporter cells of bright-field (BF; top), GFP (middle), and FM 4-64 (bottom) fluorescence images. The schematics showed abstracts of the four treatments: “MVs,” “MVs + anti-MVs,” “anti-MVs,” and “10× C16-HSL.” MVs were labeled with FM 4-64. Desalted PBS solutions containing similar concentrations of FM 4-64 were also used for MV labeling during “anti-MVs” and “10× C16-HSL” treatments as controls. (B) Scatterplots of GFP and FM 4-64 fluorescence intensities of the individual cells measured from the fluorescence images of each treatment “MVs” (red, *n* = 78), “MVs + anti-MVs” (blue, *n* = 106), “anti-MVs” (black, *n* = 85), and “10× C16-HSL” (green, *n* = 85). (C and D) Averaged fluorescence intensities of GFP and FM 4-64 on all individual cells shown in panel B, error bars show the standard errors. (E) Dynamic MV-binding processes. The time courses of FM 4-64 fluorescence intensities of the individual cells were measured from the time-lapse live-cell imaging of MV-binding processes in the absence and presence of anti-MV antibodies. The images were captured every 20 s. The individual light red and blue curves indicate the time courses in the absence (*n* = 20) and presence (*n* = 23) of anti-MV antibodies, respectively. Solid and dashed lines indicate the averages of each time course in the absence and presence of anti-MV antibodies, respectively.

We further investigated the efficiencies of MV-mediating QS signaling at a single-cell level. For quantitative single-cell measurement of MV concentrations, isolated MVs were labeled with FM 4-64 dye. Excess dyes were removed ahead of use by desalting. Figure 2A shows bright-field images and GFP/FM 4-64 fluorescence images of the AHL reporter cells used in the four treatments—“MVs,” “MVs + anti-MVs,” “anti-MVs,” and “10× C16-HSL (2 μM C16-HSL).” In “MVs” and “MVs + anti-MVs” treatments, cells showed fluorescence signals from GFP and FM 4-64. The “anti-MVs” treatment, which contained no MVs and C16-HSL, showed background-level GFP expression in AHL reporter cells. The “10× C16-HSL”-treated cells showed GFP signals but no FM 4-64 fluorescence. We then plotted distributions of correlations between FM 4-64 and GFP fluorescence intensities from individual cells treated with these four treatments (Fig. 2B). In the “MVs” and “MVs + anti-MVs” treatments, GFP expression levels and concentrations of bound MVs (FM 4-64 intensities) in each cell showed a positive correlation as indicated by the correlation coefficients of 0.74 and 0.78, respectively. The average fluorescence intensities of “MVs + anti-MVs”-treated cells for GFP and FM 4-64 were 1.6 and 1.9 times higher than those of “MVs”-treated cells (Fig. 2CD), respectively, whereas in the “MVs + anti-MVs”-treated cells, 32% of the cells had high GFP intensities (>10,000); no cell had more than intensity values of 10,000 in the “MVs” treatment. Importantly, the cells with high GFP intensities had high FM 4-64 intensity ranks in the top 50% in all cases. These results demonstrated that direct MV-binding to cell surfaces raised the efficiency of QS-signal transportation processes.

According to the analyses of bulk cells using the fluorometer, the “MVs + anti-MVs” treatment induced 10 times higher QS-signal efficiency than the “1× C16-HSL” treatment (Fig. 1E) despite similar C16-HSL contents in both treatments. The “10× C16-HSL”-treated cells showed that GFP intensities ranged from 330 to 13,991 (Fig. 2A) and 7,900 ± 400 on average (Fig. 2C), which corresponded to GFP intensities detected from the “MVs + anti-MVs”-treated cells (Fig. 2C). These results also showed that the antibody enhanced MV-mediated QS-signal transportation and showed 10 times more efficiency than free diffusion-based transportation in a reproducible manner. In “MVs + anti-MVs,” GFP signals from cells showing more than 3,000 FM 4-64 fluorescence intensities reached a plateau (Fig. 2B), indicating that the cells attached abundance of MVs highly expressed GFP. To assess FM 4-64 contamination, we used desalted PBS containing similar concentrations of FM 4-64 as that used for labeling MVs during “anti-MVs” treatment. FM 4-64 intensities were low (Fig. 2C and D), showing that FM 4-64 contamination can be ignored.

We further confirmed the effect of MV adsorption on the cell surface by anti-MV antibodies using live-cell imaging of the dynamic MV-binding process (Fig. 2E). We attached AHL reporter cells onto a coverslip equipped in a cell chamber for the microscope. We added FM 4-64 labeled MVs in PBS in the absence or presence of the anti-MV antibodies. Figure 2E presents the time courses of FM 4-64 fluorescence intensities of individual cells for 5 min following the addition of the FM 4-64-labeled MVs. The MV-binding velocities were significantly higher in the cells incubated with the anti-MV antibodies. The average speed of increasing FM 4-64 fluorescence intensities during the initial 1 min was 460 ± 150/min and 1,300 ± 400/min in the absence or presence of the anti-MV antibodies, respectively. Given that the anti-MV antibodies recognize both MVs and P. denitrificans cell surfaces (Fig. S1), they likely accumulate MVs onto the cell surface, functioning like cross-linkers and facilitating C16-HSL transportation from MVs to a cell. Therefore, we could use them as a tool to elicit MV adsorption onto the cell surface.

### AFM live-cell imaging of MV-binding processes to cell surfaces.

**FIG 3:**
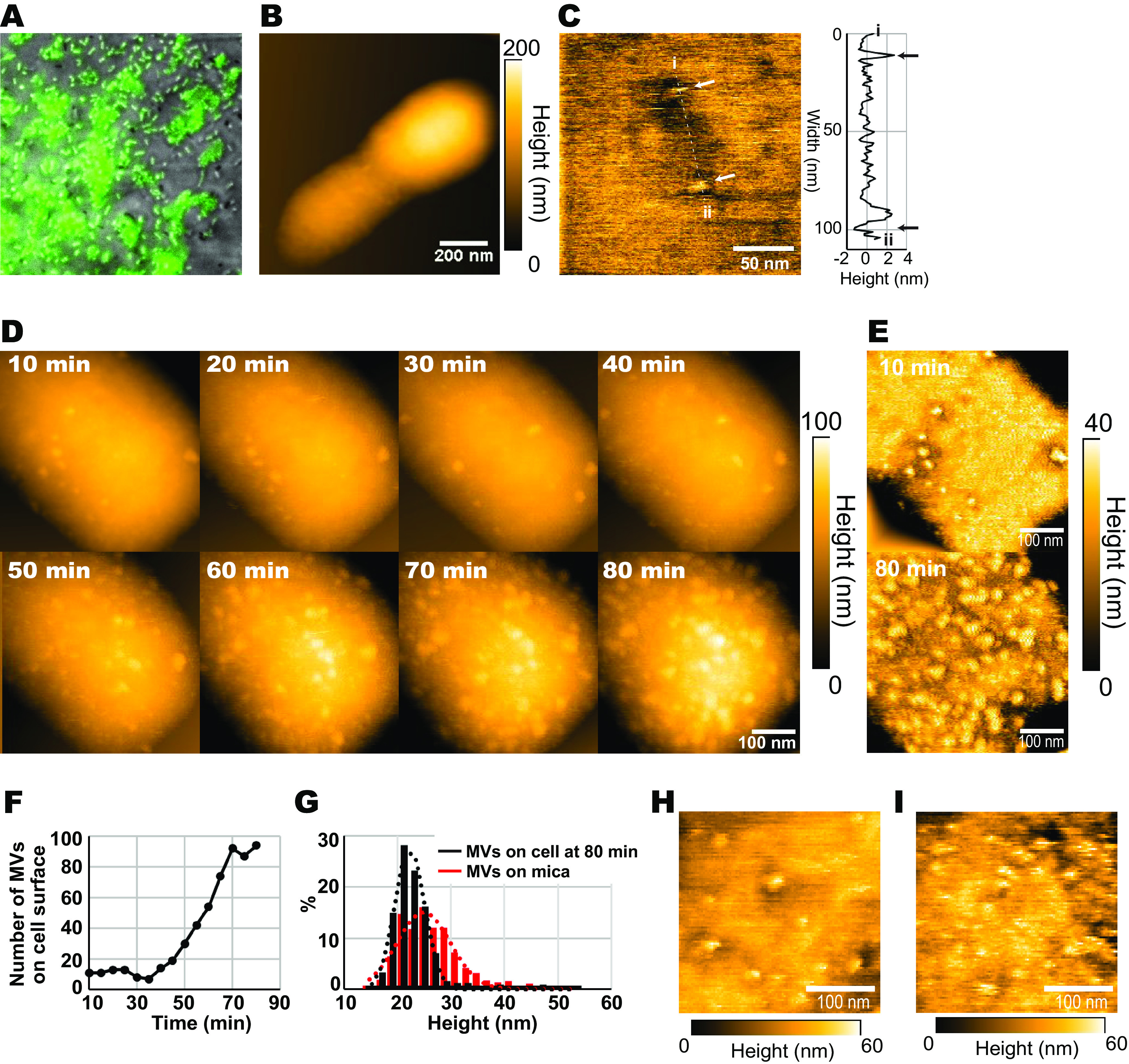
AFM live-cell imaging of the MV-binding processes on the cell’s surface. (A) Fluorescence images of AHL reporter cells immobilized on a mica surface incubated with MVs. GFP expression showed intactness of the cells and MV-mediated QS-signaling activities. (B) Low-magnification AFM topological images of an AHL reporter cell in PBS. (C) High-magnification AFM topological image of the cell’s surface (left). A line profile along the white dashed line also exists in the left panel (right). The cell’s surface appears essentially smooth. Arrows showed the tips of the small globular structures that were originally found in the P. denitrificans cell surfaces. (D) Still images of the successive AFM topological imaging movie (Movie S1) that recorded the MV-binding process to the cell surface. The images were taken after 10 to 80 min of adding MVs to the AFM-imaging chamber. (E) Flatted surface images, which were obtained by substructions of cell curvatures from the original AFM images recorded at 10 and 80 min after adding MVs. (F) The time course of the number of spherical particles found in the 500 × 500-nm area on the cell’s surface. (G) Diameter distributions of the spherical particles on the cell’s surfaces (black bars) and the isolated MVs used for this study (red bars). (H and I) Effects of adding the anti-MV antibodies during MV binding. Flatted topological images of cell surfaces treated with MVs (H) and MV plus anti-MV antibodies (I). The topological AFM images were recorded at an imaging rate for panels B, D, H, and I of 2.0 s/frame and panel C of 0.5 s/frame using panels B, H, and I with 200 × 200 pixels and panels C and D with 250 × 250 pixels.

According to the live-cell fluorescence imaging, MV adsorption to bacterial cells promotes the efficiency of QS-signal transportation. The next question is how MVs behave on/in the bacterial cell surface. Here, we imaged the MV receiving processes into living cell surfaces using high-speed AFM. Subsequently, P. denitrificans AHL reporter cells were immobilized on mica surfaces, using a method that we used previously (31, 32). The viabilities of immobilized cells were confirmed by LIVE/DEAD staining assay (Fig. S3A). Furthermore, the immobilized AHL reporter cells expressed GFP when MV-containing solutions (1.1 × 10^9^ particles/mL; same concentration for “MVs” treatment) were applied to the mica. This result indicated that MV-mediated QS-signal transportation remained active in immobilized cells (Fig. 3A).

First, we observed the AHL reporter cell surface structures. Under low-magnification images, diplococcus cells were observed (Fig. 3B and Fig. S3B). According to high-magnification observations, globular small particles were found on the cell surfaces (Fig. 3C and Fig. S3B). This structure was distributed over surfaces randomly, at approximately three to four particles in the scanning areas (300 × 300 nm). The heights of the particles also ranged from 2 to 10 nm (3.6 ± 1.5 on average [*n* = 40]) (Fig. 3C). Nevertheless, no noticeable structure larger than 10 nm was observed on the cell’s surface. This particle is proposed to be a unique structure in P. denitrificans. Previously, we were unable to observe a similar structure on the surface of the other alphaproteobacterial species *Magnetospirillum magneticum* (31) and Rhodobacter sphaeroides (32).

Additionally, for the visualization of the MV-binding process to a recipient cell, we added MVs (1.1 × 10^9^ particles/mL) into an AFM imaging chamber and then took an AFM movie of the cell’s surface structure for approximately 90 min at a 2 s/frame (Fig. 3D and Movie S1). After adding MVs, spherical structures appeared, and the number of structures increased with time. Figure 3E shows the flatted surface images obtained by substructions of the cell’s curvature from the original AFM images recorded after 10 and 80 min of the addition of MVs. Results also showed that the number of spherical structures increased after 20 min until 70 min (Fig. 3F). Likewise, Fig. 3G shows the size distributions of these spherical structures and that of the purified MVs measured by high-speed AFM on mica substrates. As shown, the corresponding two size distributions indicated that the observed spherical structures were MV bound to the cell surfaces. On the cell’s surface, MVs were bound homogeneously, inferring that no specific site for MV binding existed, but the entire cell’s surface had a homogeneous affinity to MVs. Some MVs moved laterally on the cell’s surface, finally being anchored on it (Movie S2). We have performed live-cell AFM imaging using more than 300 cells in 43 independent experiments. The MV bindings to the cell surfaces were repeatedly observed in a reproducible manner. As control observations, we monitored the cell surfaces incubated with 0.2 μM C16-HSL (“1× C16-HSL”) for 30 min (Fig. S3C) to confirm the effect of C16-HSL-dependent cellular responses in cell surfaces. We could not detect the spherical structures in the C16-HSL-treated cell surface, indicating that the spherical structures did not emerge due to cell structure alteration triggered by cells quorum sensing signaling. Moreover, long-time high-speed AFM observation did not lead to cell surface structure alterations (Fig. S4A).

Figure S4B shows the time courses of height alterations of the seven MVs binding on the cell surface during the 20–40 min after adding MVs. Although we expected that MVs could alter their structures by fusing with the cell surface, we did not find any noticeable change in the height and shape of MVs during the observations. These results indicated that the structure of MV was maintained stably after binding on the cell surface.

We further examined the influence of the anti-MV antibodies on the binding of MVs to cell surfaces using AFM. Figure 3H and I show the surface structures of cells incubated for 45 min in PBS containing 1.1 × 10^8^ particles/mL MVs without or with anti-MV antibodies, respectively. Interestingly, there was a 2-fold increase in the density of MVs when anti-MV antibodies were used. Taken together, the anti-MV antibodies facilitated the binding of MV particles to the cell surface. Nevertheless, we did not observe immunoglobulin molecules binding on the cell surfaces of the “anti-MVs”-treated cells (Fig. S5A and B). This is probably due to the impairment of the spatial resolution by the antibodies binding to the cell surfaces. The clustering of soft materials covering the sample’s surface can prevent AFM imaging.

### Dynamic shifting of MV physical properties on cell surfaces.

**FIG 4:**
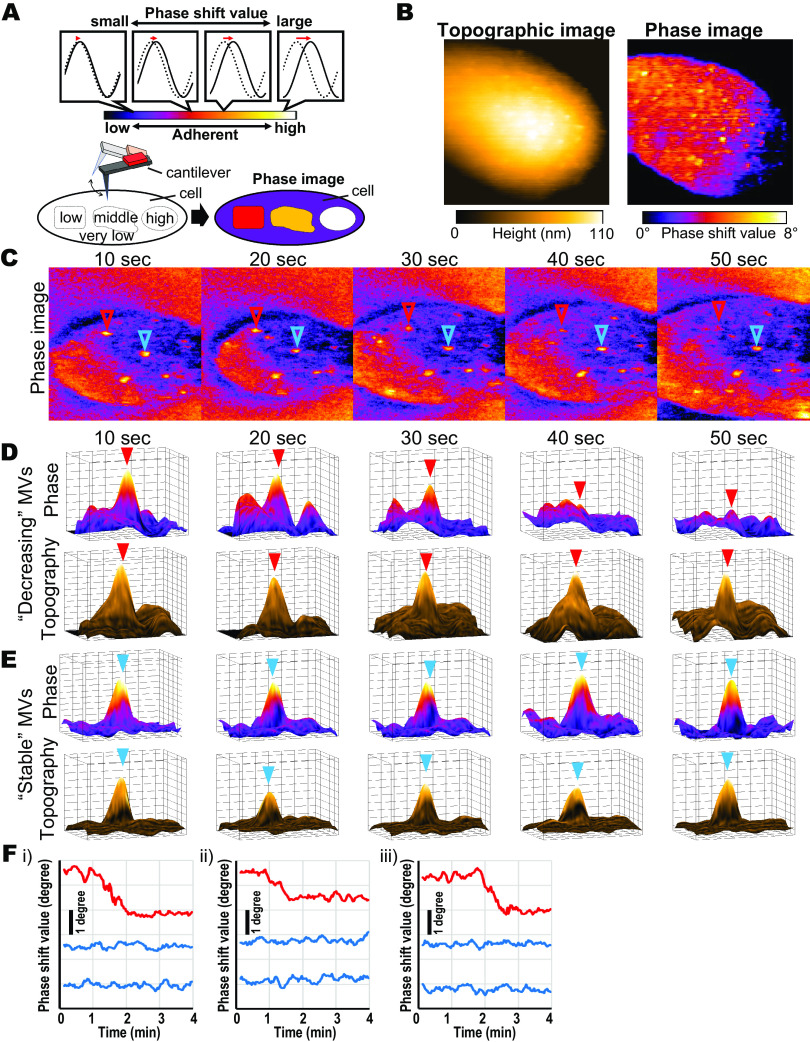
AFM phase imaging of the physical properties shifting of MVs on bacterial cell surfaces. (A) Schematic diagrams of AFM phase imaging. Wave graphs were cantilever oscillations (solid lines) and sinusoidal reference signals (dashed lines). Lengths of red arrows indicate the phase difference between a cantilever oscillation and sinusoidal reference signal. The phase difference is expressed as the phase shift degree (phase shift value). Small phase shift value indicates a low-adherent region (weak hydrophobic interaction with an AFM tip), whereas large phase shift value indicates high-adherent region (strong hydrophobic interaction with an AFM tip). (B) Topographic (left) and phase (right) images of the cell incubated with MVs at 1.1 × 10^8^ particles/mL. (C) Still images of the successive AFM phase imaging movie (Movie S3) of MV-treated cell surface. Red and blue triangles indicate “Decreasing” and “Stable MVs,” respectively. The 3D-view AFM phase (top) and topological (bottom) images of the selected area around Decreasing (D) and Stable (E) MVs are shown by red and blue triangles in panel C. (F) Time courses of three MV phase shift values obtained simultaneously in the same scanning areas. (i to iii) Time courses obtained from three independent cells. All AFM images were recorded at an imaging rate of 2.0 s/frame with 200 × 200 pixels.

The AFM phase mode allows imaging of local physical property distributions by detecting the phase shift of cantilever oscillations from that of the excitation signal (34, 35). Based on this fact, we used phase imaging techniques to measure MV physical property alterations on cell surfaces. A phase of the AFM cantilever oscillation was shifted, depending on the energy dissipation from cantilever tips to sample surfaces (Fig. 4A). Since the main source of this energy dissipation was a hydrophobic adhesion force between the sample’s surface and AFM cantilever tip during high-speed AFM imaging in liquids (39), it enabled hydrophobicity mapping of the sample surface. The bacterial culture contains 10^8^ to 10^9^ MV particles/mL (19, 40). Therefore, we added MVs into the AFM imaging chamber to 1.1 × 10^8^ particles/mL to observe the MV-binding process in near-to-culture conditions using phase imaging. Figure 4B shows the topographic and phase images simultaneously obtained from the AHL reporter cell incubated 50 min after MVs were added. In phase images, small phase shift values (purple) indicated low adherence, whereas high phase shift values (red to yellow) indicated high adherence. Phase images showed that MVs on cell surfaces had a higher phase shift value than cell surfaces, indicating that bound MVs were more adherent to the AFM cantilever tip than cell surfaces. Additionally, MV phase shift values were different from those obtained from the cell’s surface and ranged from 1.8 to 5.4 degrees. This dispersion of MV phase shift value was consistent with that obtained for P. denitrificans MVs in a previous report (36). In contrast, although we observed the “MVs + anti-MVs” and “anti-MVs”-treated cell surfaces using phase imaging, the phase images did not reveal noticeable structures (Fig. S5C and D). The overall binding of the anti-MV antibodies to the cells can be attributed to the uniformity of their physical properties on cell surfaces. Hence, we were unable to measure the phase shift values of MVs on antibody-treated cell surfaces.

Next, we focused on the dynamic alteration of MV phase shift values on cell surfaces. Notably, the phase of some MVs shifted during successive observations (Fig. 4C and Movie S3). Interestingly, the height of the MVs remained unchanged when there was a decrease in their phase shift values (Fig. 4D). The reduction in MV phase shift values continued until it reached the same level as that of the cell surface (Fig. 4D and Movie S4). Moreover, the phase shift values of the other MVs observed simultaneously in the same scanning area were constant (Fig. 4E and Movie S4). Figure 4F shows the time courses of the phase shift value of three MV particles simultaneously obtained in the same scanning areas from the three independent bacterial cells. The decrease in phase shift values occurred in particular MV particles and did not correlate with the alteration of phase shift values of the other MV particles in the same scanning areas. Hence, we termed MVs with decreasing and constant phase shift values as “Decreasing” and “Stable” MVs, respectively.

### Analysis of the physical behavior of a single MV particle in the cell surface.

**FIG 5:**
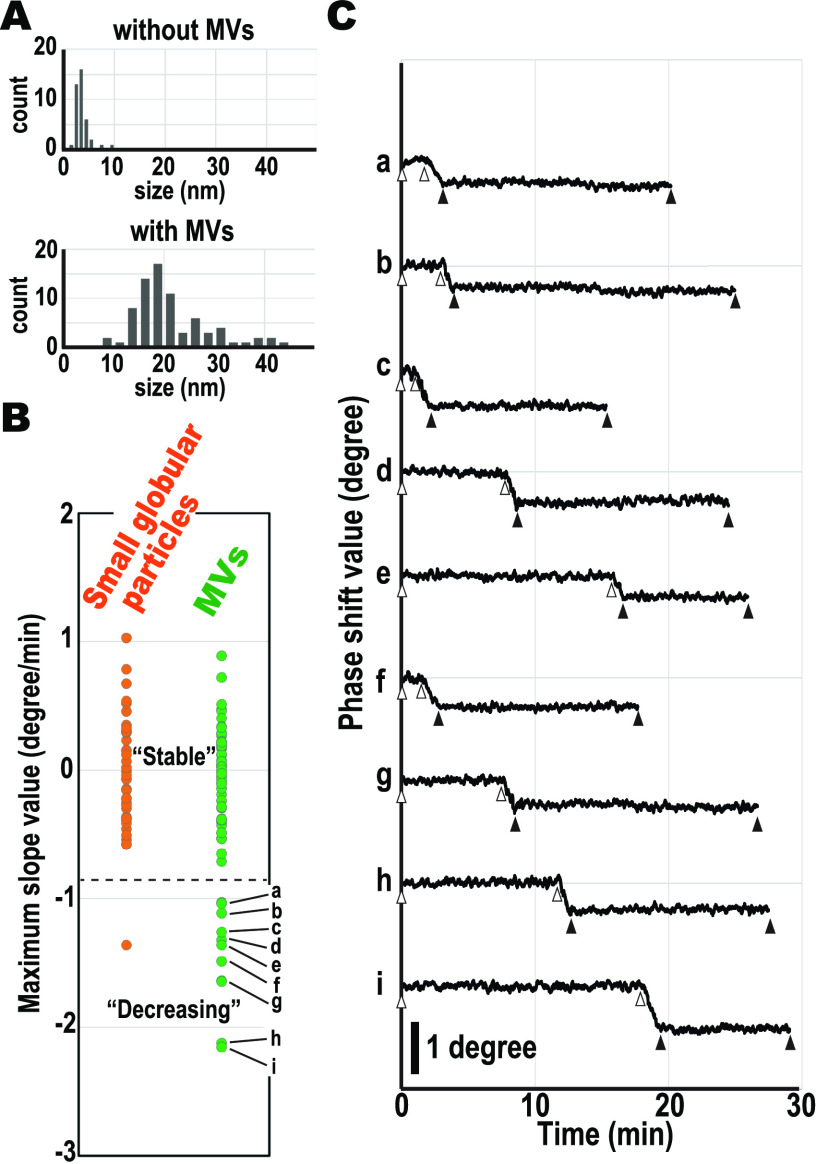
Single particle analysis of dynamic phase shift value alternation. (A) The size distributions of the particles found in cells incubated without (*n* = 40) and with (*n* = 76) MVs. The average sizes of these particles found in cells without and with MVs were 3.6 ± 1.5 nm and 21.6 ± 7.6 nm, respectively. (B) The distributions of the maximum slope values of the phase shift time courses of the small globular particles (orange) and MVs (green). (a to i) Decreasing MV particles with maximum slope values less than −1 degree/min. (C) The phase shift value’s time course of the nine Decreasing MVs shown in panel B. The phase shift values before (between open triangles) and after (between solid triangles) the decreasing events were constant.

We found that the phase shift values of a portion of the MV particles dynamically decreased during the observations. We confirmed whether the decreasing phase shift values are an MV-specific phenomenon or not. For this, we tested the time courses of the phase shift values of 40 globular small particles originally found in 12 cells incubated without MVs and 76 particles from 9 cells incubated with MVs. Almost all the particles in the MVs cell surfaces incubated with MVs were significantly larger than the globular small particles (Fig. 5A); hence, these were recognized as MVs. We estimated the maximum slope values for each particle’s time course (*see* Materials and Methods section). Figure 5B shows the distributions of the maximum slope. The slope value distribution of the globular small particles ranged from −0.58 to 1.03 to −0.03 ± 0.40 on average, whereas the slope value distribution of MV ranged from −2.76 to 0.89, −0.2 ± 0.6 on average. In the maximum slope distribution of MVs, nine particles (Fig. 5B, a to i) showed less than −1 degree/min and were termed Decreasing MVs, while the others were Stable MVs. Table 1 shows the properties of these nine Decreasing MVs.

**TABLE 1:** Parameters of individual “Decreasing” MVs^*a*^

MVs	a	b	c	d	e	f	g	h	i	Averages^*b*^
ΔPhase shift value (degree)	−1.00	−1.06	−1.81	−1.44	−1.01	−1.39	−1.07	−1.22	−2.11	−1.3 ± 0.4
Maximum slope value (degree/min)	−1.03	−1.26	−1.32	−1.36	−1.49	−1.64	−1.65	−2.12	−2.16	−1.6 ± 0.4
Size (nm)^*c*^	16.9	24.5	23.5	21.3	19.3	25.8	22.3	28.2	18.2	−22 ± 4
Dwell time (sec)^*d*^	124	186	66	424	974	102	470	720	1,092	460 ± 400

a The alphabets a to i indicate MVs labeled in Fig. 5B.

b The averages and standard deviations of values obtained from MV (a to i).

c Size is the average value for 20 s before the initiations of decreasing phase shift values.

d Dwell times show the time periods from the starting of AFM observations to the initiation of decreasing phase shift values.

Figure 5C shows the entire time course of the phase shift values from the start of the AFM recording of the nine Decreasing MVs. Each time course demonstrated an irreversible decrease in the phase shift values. This decrement occurred once, and no Decreasing MVs were detected to have a second decrease. The maximum slope values were from the Decreasing MV time courses raised from this type of dynamic phase decreasing. Conversely, the time courses of the Stable MVs were relatively flat (Fig. S4E). The maximum slope values obtained from Stable MV time courses were nearly zero and ranged from −0.71 to 0.89, −0.01 ± 0.34 on average. Moreover, the time courses of Decreasing MV before and after the decreasing events were constant (Fig. 5C; between open and closed triangles, respectively). Hence, the phase shift value of MV after the decreasing event appeared to have reached an equilibrium state. Additionally, because the phase shift values of purified MVs were stable during observation on the mica’s surface for more than 60 s (36), this shifting of phase shift value, i.e., shifting of hydrophobic adhesion, occurred on the cell’s surface specifically. Taken together, these results indicated that the event of the decrease in the phase shift value occurs specifically in the MVs attached to the cell surface.

## DISCUSSION

**FIG 6:**
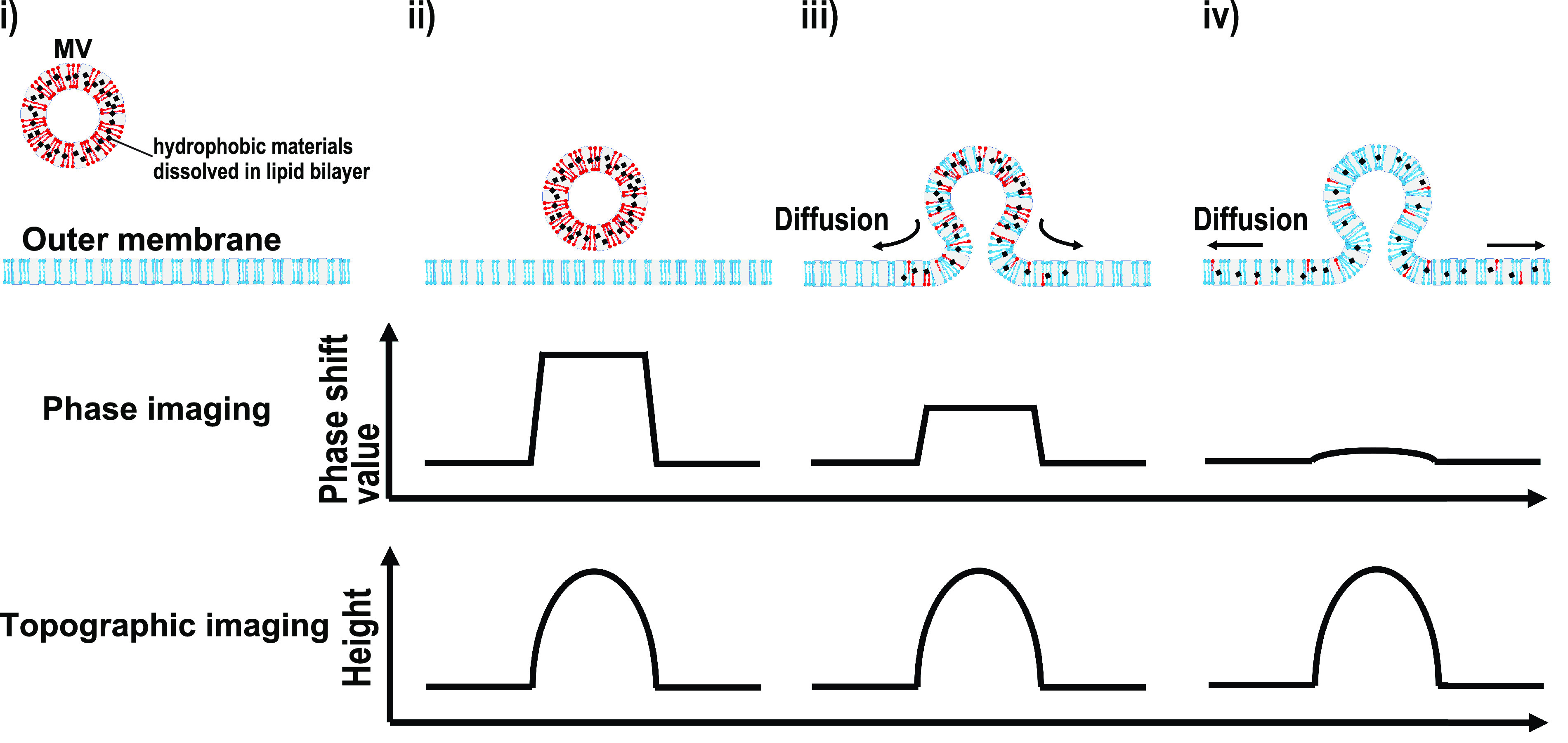
Model for MV fusion into cell surface. (i) Before MV binding on a cell’s surface. The compositions were different between MV and cell’s outer membrane. MVs contained hydrophobic materials, including C16-HSL in the hydrophobic region of phospholipids. (ii) MV is adsorbed to a cell’s surface by electrostatic interactions. Since MV and the outer membrane are separated, the MV phase shift value was higher than that of the outer membrane’s surface. (iii) MV fusing with the outer membrane. The hydrophobic components, containing lipids, are exchanged between the MV and outer membrane through diffusive movements. (iv) The composition of MV is similar to that of the outer membrane.

Bacterial cell surfaces function as interfaces for extracellular signals, stresses, and environmental circumstances. This study is the first to elucidate the measurements of the local dynamic physical property-shifting processes in living cell surface structures. Combining live-cell and phase imaging techniques using high-speed AFM enabled the achievements of our study. Because phase shift values mainly showed surface hydrophobicity, thereby attributing chemical compositions to the sample’s surface (39), a shift in the MV phase shift value indicates exchanging components consisting of MVs on the cell surface. Figure 6 is a model proposed for MVs received into Gram-negative bacterial cell surfaces. As shown, (i) MV approached the outer surface of a bacterial cell’s membrane by simple diffusion through the extracellular milieu. (ii) MVs are then attracted and bound by interactions based on electrostatic and London-van der Waals forces (17, 21). These interactions proposed a contribution to the species-specific binding of MVs in Gram-negative bacteria (21). At this stage, AFM recognizes the binding of MV to the cell’s surface in both phase and topographic images. MV is proposed to just be attached to the cell’s surface but not fused with the outer membrane (Stable MVs). (iii) However, MV membranes can also be fused with the outer membrane (Decreasing MVs). This step occurs randomly in the MVs bound on the cell surface. By membrane fusion, the hydrophobic cargo molecules, including hydrophobic QS-signal molecules in MVs, thus start to diffuse into the outer membrane. (iv) Subsequently, the diffusion of hydrophobic materials continues. Decreasing the phase shift value suggests a diffusion of hydrophobic components from the MVs to cell surfaces. The phase shift value therefore became a constant after the decreasing event, inferring that the diffusion reached equilibrium (Fig. 5C).

According to the AFM observation, Decreasing MVs have been found at ~2.4 particles/h in the scanning area (400 × 400 nm). Given that this area corresponds to approximately 7% of the entire cell surface area, the results indicate that ~34 MV phase-decreasing events could occur on the single-cell surface in 1 h, which means that the phase decreasing of MV particles occurs frequently on cells growing under culture conditions. Moreover, we can be fairly certain that this event occurs in bacterial cells living in natural environments (e.g., biofilms) that contain MVs as major components (41, 42).

Surprisingly, MV heights were unaltered during the fusion and diffusion processes (Fig. 4). This is proposed to be due to the outer membrane’s environment that is closely packed by clustered outer membrane proteins (OMPs) (31, 43, 44) and liquid-crystalline state of lipopolysaccharides (45). According to AFM imaging, living cell outer membranes are covered by OMPs clusters and LPS patches in E. coli (43), whereas OMP’s cluster diffusion coefficients showed slow diffusion rates (3.2 ± 0.4 nm^2^/s), in *M. magneticum* AMB-1 (31). Hence, MV contains significant concentrations of membrane proteins that are proposed not to be inserted and diffused into the crowd outer membrane even after MV fusion.

MV-mediated C16-HSL transportation showed higher efficiency than the simple diffusion of soluble free C16-HSL (Fig. 1 and 2). Because an error in measuring C16-HSL concentration was about 2% (19), the calculation of C16-HSL probably not have affected the results. One of the advantages of MV mediating signal transportation is that one MV particle enables the transportation of quantitatively sufficient C16-HSL signal to induce C16-HSL signal-dependent gene expression in a cell (19). In the “1× C16 HSL” treatment, a portion of cells might not have been able to obtain a sufficient amount of C16-HSL by simple diffusion. In addition, the polyclonal antibodies produced against P. denitrificans MVs enhanced the efficiency of the MV-mediated QS-signal transportation (Fig. 1 and 2). As expected, antibody generation against pathogenic bacteria-derived MVs or those derived from nonpathogenic residents in their hosts, and the enhancement in MV transportation efficiency due to these antibodies may be a common occurrence in the body. Hence, the occurrence and importance of antibody-mediated MV-receiving enhancement *in vivo* are worthy of further studies.

Recently, Bos et al. observed the dynamic behavior of MVs that were more than 200 nm in diameter in E. coli microcolonies using fluorescence microscopy (24). They imaged the dynamic “sliding,” “free,” and “bound” states of MVs and found that the MVs spent most of their time moving at the bacterial membrane-medium interface. They proposed that the proximal motion of MVs to the cells favors communication between these cells. Unfortunately, it was difficult to capture the motion of MVs, such as sliding and proximal movement to their cell’s surface, using AFM as this technique was unable to image loosely associated structures on solid surfaces. This limitation is proposed to be the reason why no large MVs with a diameter of ≥100 nm were observed in this study. Alternatively, a majority of MVs (~80%) were small, 20 to 40 nm in diameter (Fig. 3G), which were difficult to image using light microscope-based methods. However, we were still able to observe the behavior of major MVs. It was observed that the MV structures were stably maintained on the cell surfaces following their fusion with the outer membrane. This finding coincided with the binding states of MVs that had been observed repeatedly under transmission electron microscopy (TEM) (17, 18, 21).

A major advantage of phase imaging is that it can facilitate obtaining information on surface physical properties instantly and simultaneously via capturing the topological images within the same imaging areas. Because the imaging technique for measuring local dynamic physical property-shifting processes on living cell surface structures is still developing, our knowledge about how cell surfaces work as bacterial interfaces remains limited. Hence, we believe that high-speed AFM live-cell imaging provides a novel approach to analyzing life phenomena occurring in bacterial cell surfaces and contributes to the understanding of the functioning of cell surfaces as bacterial interfaces.

## MATERIALS AND METHODS

### Bacterial strains, plasmids, and growth condition.

Paracoccus denitrificans (Pd1222) and AHL reporter strain of P. denitrificans were used in this study. P. denitrificans strains were grown routinely in tryptic soy broth (TSB) (46), to which 50 μg/mL kanamycin had been added, if necessary, at 30°C with shaking. The AHL reporter strain was constructed in a previous study (19) containing the AHL reporter plasmid (pPL*las*) (38) and lacking *pdnI* encoding AHL syntheses. pPL*las* plasmid is engineered from the components of las promoter in the QS-system from Pseudomonas aeruginosa, which responds to long-chain AHLs containing C16-HSL. We additionally knocked out a gene encoding a putative extracellular matrix synthesis protein (in preparation) in the AHL reporter strain to prevent forming cell aggregations that cause hampering AFM imaging.

### MV purification and quantification.

MVs were purified from wild-type P. denitrificans or AHL reporter strain cell cultures grown for 24 h. Spent cultures were obtained by centrifugation for 10 min at 6,000 × *g* at 4°C. Afterwards, the supernatant was filtered through a 0.45-μm pore-sized mixed cellulose ester filter. Subsequently, the filtered spent culture was ultracentrifuged for 1 h at 150,000 × *g* and a temperature of 4°C, after which the pellets were resuspended in PBS. Finally, the concentration of MVs obtained after the resuspension was measured using NanoSight NS300 (Malvern Instruments Ltd., Malvern, UK).

### Generation and purification of polyclonal antibodies against MVs.

We generated polyclonal antibodies against P. denitrificans MVs by surgically implanting a purified MV suspension aliquot (1.1 mg proteins/mL) as an antigen in rabbits. Antibodies were purified from the antiserum using protein-A binding columns (Dojindo, Kumamoto, Japan). For negative-control experiments, antibodies were purified from the preimmune serum. The purified antibodies (13 μg/mL, final concentration) were mixed with MVs and incubated for 1 h at room temperature before being used in the experiments. The protein concentration of the purified anti-MV antibodies was measured using the Pierce BCA protein assay kit (Thermo Fisher Scientific, Waltham, MA). For titration with the anti-MV antibody, C16-HSL was diluted in PBS containing 13 μg/mL of the purified anti-MV antibody.

### Transmission electron microscopy.

We conducted imaging of the negatively stained samples using a JEOL JEM-2100Plus TEM (JEOL, Tokyo, Japan). The TEM was operated at 120 kV in a bright-field mode. The immunogold-staining of P. denitrificans MVs was performed as previously described (47) with slight modifications. Briefly, the grids with the purified MVs were rinsed thrice, each for 1 min, on a drop of PBS, and subsequently incubated with the antiserum or preimmune serum for 1 h at room temperature. The sera were diluted at 1:25 with PBS. After being rinsed three times with PBS, each for 1 min, the specimens were incubated with 5-nm or 15-nm diameter gold-conjugated goat anti-rabbit IgG (EY laboratories Inc., San Mateo, CA) diluted at 1:50 for 1 h at room temperature. After being rinsed three times with PBS for 1 min, the specimens were then rinsed with deionized water twice for 3 min each time. Following immune staining, the specimens were negatively stained with 2% uranyl acetate.

### Fluorometer measurements.

For this analysis, GFP’s expression of the P. denitrificans AHL reporter strain was measured using a fluorescence spectrophotometer; Shimadzu RF-5300PC (Shimadzu, Kyoto, Japan). AHL reporter cells were then cultured until they reached the late log phase. Subsequently, the cultures were adjusted at an absorbance at 600 nm to ca 1.0 and aliquoted. Later, MVs, a mixture of MVs and anti-MV antibodies, C16-HSL, and anti-MV antibodies were added to each aliquot and incubated with shaking at 30°C for 3 h. The final concentration of MVs was 1.1 × 10^9^ particles/mL, that of anti-MV antibodies was 13 μg proteins/mL, and that of C16-HSL was 0.2 μM. The GFP emission spectra of the cell suspensions excited at 488 nm were obtained at room temperature.

For titration, we used C16-HSL, *N*-hexadecanoyl-l-homoserine lactone (Cayman Chemical, Ann Arbor, MI), which was dissolved (5 mM C16-HSL) in dimethyl sulfoxide and stored at −20°C until use. C16-HSL was diluted in PBS, adjusted to given C16-HSL concentrations ([C16-HSL]), and added to AHL reporter strain cultures under the same above-described experimental conditions. The GFP emission spectra of the cell suspensions containing 5, 3, 2, 1, 0.5, 0.3, 0.2, and 0.1 μM C16-HSL were measured three times each, and GFP fluorescence intensities (emission = 515 nm) were measured. The standard curve of [C16-HSL] versus GFP fluorescence intensity was obtained using the fitting of a logarithmic equation:

y=137.35ln(x)+460.68

where *y* is the GFP fluorescence intensity and *x* is [C16-HSL], which was the required concentration for similar levels of GFP expression. The QS-signaling efficiency was estimated based on this equation. For titration with the anti-MV antibody, C16-HSL was diluted in PBS containing 13 μg/mL of the purified anti-MV antibody.

### Fluorescence imaging.

We used highly inclined and laminated optical sheet (HILO) microscopy (48). The imaging setup was based on a total internal reflection fluorescence (TIRF) microscope system with an inverted microscope (Nikon, Tokyo, Japan), equipped with a 100× CFI Apo TIRF lens objective and a ×1.5 C-mount adapter. Also, 488-nm and 562-nm lasers (Sapphire; Coherent, Santa Clara, CA) were used to illuminate the sample at an inclined angle, slightly steeper than the critical angle required for a total reflection to illuminate an entire bacterial cell.

To analyze the correlations between adhering MV concentrations and GFP fluorescence intensities (Fig. 2), images were acquired using a high-sensitivity electron-multiplying charge-coupled-device camera (iXon3; Andor, Belfast, Northern Ireland) with EM and preamplifier gains of 296 and 2.4×, respectively. The exposure times for bright-field, GFP, and FM 4-64 images were 10, 300, and 900 ms, respectively. For sample preparation, AHL reporter cell cultures were prepared using the same method as fluorometer measurements and adjusted to the absorbance at 600 nm of ca 1.0. MVs were stained with an FM 4-64 dye (Thermo Fisher Scientific, Waltham, MA). MV solutions (2.2 × 10^10^ particles/mL) were incubated with the same amount of the FM 4-64 solution (2 μg/mL) for 30 min, after which excess FM 4-64 was removed using Zeba Spin Desalting Columns (Thermo Fisher Scientific, Waltham, MA). Subsequently, FM 4-64-labeled MVs (final concentration of 1.1 × 10^9^ particles/mL) were mixed with AHL reporter cell suspensions with or without the purified anti-MV antibodies (13 μg/mL) for “MVs” and “MVs + anti-MVs” treatments, respectively. The cultures were incubated for 3 h at 30°C with shaking. For “anti-MVs” and “10× C16-HSL” treatments, the purified anti-MV antibodies (13 μg/mL) or 2 μM C16-HSL were also dissolved in PBS, mixed with AHL reporter cell suspensions, and then incubated for 3 h at 30°C with shaking. In these cases, the anti-MVs and C16-HSL solutions were premixed with 1 μg/mL FM 4-64 and then desalted using Zeba Spin Desalting Columns to assess the possibility of FM 4-64 contamination. The treated cells were put onto slide glasses and placed on coverslips. Next, we microscopy took the bright-field, GFP, and FM 4-64 images of these samples using HILO microscopy. The images were processed using the NIS Elements AR (Nikon, Tokyo, Japan) and ImageJ software. GFP and FM 4-64 fluorescence intensities of each cell were also obtained from each image using the ImageJ “measurement” command. In this study, the fluorescence intensity of each cell was defined as the intensity per unit area, calculated by dividing the total fluorescence intensity of the area of a cell obtained from bright-field images.

Time-lapse imaging of the MV-binding process was performed in an Attofluor Cell Chamber (Thermo Fisher Scientific, Waltham, MA) incubated at 30°C using an incubation system for microscopes (Tokai Hit, Fujinomiya, Japan). The cells were attached to a poly-l-lysine-coated coverslip. Upon the start of time-lapse imaging, the FM-4-64-labeled MVs solution (1.1 × 10^9^ particles/mL) was added to the chamber. The exposure times for FM-4-64 and bright-field images were 500 and 10 ms, respectively, at 20-s intervals. The samples were illuminated only during exposure.

Immobilized cell viabilities on mica substrate were also assessed using the LIVE/DEAD BacLight bacterial viability kit (Thermo Fisher Scientific, Waltham, MA). After the immobilized cells were washed, the fluorescent reagent mixture was put on mica and incubated for 15 min. This setup was then observed for green (live cells) and red (dead cells) fluorescence.

For immunofluorescence stainings, AHL reporter cells were attached to a poly-l-lysine-coated coverslip set in an Attofluor Cell Chamber. The cells were washed three times with 1 mL of 1% BSA in PBS and then incubated with the antiserum or the preimmune serum for 1 h at room temperature. The sera were diluted at 1:50 with 1% BSA in PBS. After being rinsed with 1 mL of 1% BSA in PBS five times, each for 1 min, the specimens were incubated with Alexa Fluor 488 conjugated goat anti-rabbit IgG (Thermo Fisher Scientific, Waltham, MA) diluted at 1:100 with 1% BSA in PBS for 1 h at room temperature. After being rinsed five times with 1% BSA in PBS for 1 min each, bright-field and Alexa Fluor 488 images of these samples were taken using HILO microscopy. The bright-field and Alexa Fluor 488 image exposure times were 100 and 30 ms, respectively.

### The high-speed AFM setup.

For this analysis, we used a laboratory-built tapping mode coupled with a high-speed AFM apparatus (25). For phase imaging, the phase shift between the cantilever oscillation and excitation signal was detected using a lock-in-amplifier (HF2LI; Zurich Instruments, Switzerland). The small silicon-nitride cantilever (BL-AC7-DS; Olympus, Tokyo, Japan) had a spring constant *k* ~0.2 N/m and a resonance frequency *f *= 600 to 800 kHz in water. The probe tip was also grown on the top of the cantilever by two times electron beam depositions using an electron beam lithography system (ELS-7500UK; Tokyo, Elionix, Japan). The total deposition time was 180 s. Then, the free-oscillation peak-to-peak amplitude of the cantilever (A_0_) was set at 5 nm, and the amplitude set point was at 0.75 to 0.85 of A_0_.

### Preparation of AFM specimens.

Live-cell AFM imaging was performed as described previously (31, 32). Briefly, we placed 0.1% poly-l-lysine onto the mica and placed them in an oven, after which we allowed it to dry for 3 h at 60°C. The P. denitrificans AHL reporter cells were cultured in a 5 mL TSB medium and grown until their absorbance (600 nm) reached 0.6 to 0.7. The cultures were then centrifuged for 5 min at 8,000 × *g*, after which cell pellets were suspended in PBS. After being rinsed twice with PBS, the cell pellets were resuspended in 200 μL PBS. Then, the resulting suspension was applied to the poly-l-lysine-treated mica. Subsequently, the stage was incubated for 30 min in a moist chamber. Afterward, the stages were rinsed with PBS. After that, bacterial cell surfaces were imaged using AFM.

To observe the MV-binding process, the MV suspension was added to the AFM imaging chamber (54 μL in volume) during AFM’s imaging of cells. First, 9 μL PBS buffer was removed from the edge of the chamber using a gel loading tip equipped to a pipette, and then 9 μL of MV suspension was added. The final MV concentration was 1.1 × 10^9^ particles/mL. For long-time successive AFM imaging to record MV-binding process into cell surfaces, AFM images were taken at 2 s/frame for approximately 80 min. Scanning areas were 500 × 500 nm with 250 × 250 pixels. Low-magnification AFM images were recorded at 2 s/frame and a scanning area of 1,200 × 1,200 nm with 200 × 200 pixels. High-magnification AFM images were recorded at 0.5 s/frame and a scanning area of 250 × 250 nm with 250 × 250 pixels.

For phase imaging, AHL reporter cells were immobilized on a poly-l-lysine-treated mica as described earlier. Then, the MV suspension (1.1 × 10^8^ particles/mL) was loaded onto the mica and incubated for 45 min at room temperature. Subsequently, the sample was rinsed with 100 μL of PBS to wash out unbound MVs and used for AFM observations. AHL reporter cells incubated without MVs immobilized on mica were observed to obtain successive phase imaging of small particles in cell surfaces. The successive AFM phase imaging was performed for 15 to 30 min for individual cell surfaces. The AFM phase images were recorded at 2 s/frame in a scanning area of 400 × 400 nm with 200 × 200 pixels.

### Analysis of AFM images.

Images were processed using the ImageJ software. Linear adjustments to signal contrast/brightness were made in the images presented, but no gamma settings were changed. We applied a flattening filter to the successive topographic and phase images to remove the substrate-tilt effect using a “fit polynomial” plugin. Bacterial cell surfaces are highly curved. We prepared a flattened surface image to clearly show the cell surface structures. The outlines of cells were subtracted from those of the topographic images using the “subtract background” command in ImageJ to obtain the flattened cell surface images. The MV diameters were also obtained by measuring heights at the top of each MV in the obtained flattened surface images.

The phase shift values of each particle found on the cell surface were estimated as follows. The phase shift degree obtained at the top of each particle was used to avoid artifacts from parachuting (36). The top positions were obtained as the “max gray value” of each particle by the “measure” command in ImageJ. Phase shift values of particles in cell surfaces were estimated by differences between the phase shift value of the top of the particle and the background (the averaged phase shift values of proximal cell surface area). The 60 × 60-nm area around the particle was used as the proximal cell surface area.

To compare the dynamics of physical property, we prepared the time courses of phase shift values from each particle. The time courses for phase shift value alteration of the top of each particle were plotted from each frame of the AFM movies. Then, we calculated the slope values of fitting lines for a 30-s time span in each time course. The 30-s fitting time span was scanned with 2-s steps from the start point to the endpoint throughout the time course. The maximum slope value obtained from a single time course was used for the representative slope value of the particle.
