# Supplementary material for: Physical Properties and Shifting of the Extracellular Membrane Vesicles Attached to Living Bacterial Cell Surfaces
Source: Microbiol Spectr. 2022 Nov 16;10(6):e02165-22. doi: 10.1128/spectrum.02165-22 (PMC9769862; doi:10.1128/spectrum.02165-22)
Supplement: Supplemental file 5 — Fig. S1 to S5 and legends of Movies S1 to S4. Download spectrum.02165-22-s0005.pdf, PDF file, 6.6 MB [file spectrum.02165-22-s0005.pdf]

Supporting Information for

Physical properties and shifting of the extracellular membrane vesicles attached to living bacterial cell surfaces

Yousuke Kikuchi, Masanori Toyofuku, Yuki Ichinaka, Tatsunori Kiyokawa, Nozomu Obana, Nobuhiko Nomura*, Azuma Taoka*

*Corresponding author. Email: nomura.nobuhiko.ge@u.tsukuba.ac.jp (N.N.); aztaoka@staff.kanazawa-u.ac.jp (A.T.)

This PDF file includes:

- Supplementary Figure Legends
- Supplementary Figures: Figures S1 to S5
- Supplementary Movie Legends for Movies S1 to S4

Other Supplementary Materials for this manuscript include the following:

Movies S1 to s4

Supplementary Figure Legends

FIG S1 Transmission electron microscopic images of immunogold-stained *P. denitrificans* MVs with anti-MVs serum (A and B) and preimmune serum (C and D). Arrowheads indicate 15-nm (A and C) and 5-nm (B and D) gold particles. Each gold particle represents a complex of antigen, rabbit IgG, and anti-rabbit IgG antibody-gold conjugate. The gold particles specifically localized the MVs to anti-MVs antibodies (A and B). In contrast, significantly fewer gold particles could be observed at random positions in the negative control experiment using preimmune serum (C and D). (E and F) Immunofluorescence-staining of the AHL reporter cells labeled with (E) anti-MVs and (F) preimmune serum. Each fluorescence and bright-field image in panels E and F were acquired under the same microscopic conditions. Cell-bound IgGs were labeled with anti-rabbit IgG antibody Alexa Fluor 488 conjugates. The fluorescence signals were detected from the peripheral region of the cells, suggesting that the anti-MVs antibodies were bound to the cell surface. The immunogold-staining (A-D) and the immunofluorescence-staining (E and F) indicated that the anti-MVs antibodies recognize both the MV and *P. denitrificans* cell surfaces.

FIG S2 (A) Effects of C16-HSL-free MVs addition. Representative fluorescence spectra used as data for Fig. 1F. Fluorescence spectra of the AHL reporter cell suspensions incubated with MVs (1.1×10^9 particles/ml; “MVs” treatment, red), “MVs” treatment plus C16-HSL-free MVs (1.1×10^{10} particles/ml, light red), 0.2 μM C16-HSL (“1 × C16-HSL” treatment, green), and “C16-HSL” treatment plus C16-HSL-free MVs (1.1×10^{10} particles/ml, light green). (B) Fluorescence titration spectra of the AHL reporter cell

suspensions incubated with purified anti-MVs antibodies-containing PBS (final concentration of 13 μg/ml) and C16-HSL ranging between 0.1–5 μM. (C) Relationship between C16-HSL concentrations and GFP fluorescence intensities in the presence of the anti-MVs antibodies estimated from fluorescence spectra in panel B (black). GFP fluorescence intensity values observed from emissions at 515 nm. The data are represented as the means ± SE (bars) (n = 3). The equation of the fitted curve is indicated. The green squares and the equation represent the relationship between C16-HSL concentrations and GFP fluorescence intensities without anti-MVs antibodies are highlighted in Fig. 1D for comparison. (D) Fluorescence spectra of the AHL reporter cell suspensions incubated with MVs plus anti-MVs antibodies ("MVs + anti-MVs" treatment, solid line) and the flow through of Protein-A Sepharose column (dashed line).

FIG S3 (A) Immobilized cell viability on a mica substrate assessed using the LIVE/DEAD BacLight bacterial viability kit. (Ai) Bright-field image of immobilized cells on a mica substrate. (Aii) Merged green and red fluorescence images of stained cells using the LIVE/DEAD BacLight bacterial viability kit. Almost all cells are stained in green fluorescence on the mica substrate. Live bacteria with intact membranes fluoresce in green, while those with damaged membranes in red. (B) AFM images of *P. denitrificans* cell surface in PBS. (Bi) Low-magnification images of *P. denitrificans* cells. (Bii and iii), High-magnification images of *P. denitrificans* cell surfaces. Arrowheads indicate globular small particles observed with non-treated cell surfaces. (C) Effect of the cell surface structure on the C16-HSL treatment. AFM images of *P. denitrificans* cell surface incubated with (Ci) MVs-containing PBS (1.1×10^9 particles/ml), (Cii) PBS, and (Ciii) 0.2 μM C16-HSL-containing PBS. The AFM imaging was performed 30 minutes

after the incubations. The line profiles between a to b in each AFM image are indicated on the light sides. The AFM images were recorded at an imaging rate of 2.0 sec/frame and 200 × 200 pixels for low-magnification images in panel Bi, and at imaging rates of 0.5 sec/frame and 100 × 100 pixels for high-magnification images in panels Bii, Biii, and C.

FIG S4 (A) Still images of the long-time successive AFM topological images of *P. denitrificans* cell surfaces in PBS without MVs. The AFM image was taken for 60 min at an imaging rate of 2.0 sec/frame. Long-time AFM imaging did not cause any apparent damage to the cell surface structure. The line profiles in each AFM image are highlighted at the bottom of each image. (B) Time-dependent structural alterations of MVs attached to the cell surface. The time courses of the height of seven MVs between 1–7. (C) Four MVs (1–4) were recorded from 40 min to 80 min, and (D) three MVs (5–7) were recorded from 60 min to 80 min after adding MVs (Movie S1). All heights of MVs on the cell surface were stable. We were unable to observe alteration of MV heights during the AFM observation on the cell surface. (E) The time courses of the representative MV phase shift values throughout the AFM observations. i), ii), and iii), iv) are time courses from "Decreasing" and "Stable" MVs, respectively. The maximum slope values in the time courses are highlighted. In i), and ii), the phase shift values before (between open triangles) and after (between solid triangles) the decreasing phase shift value were constant.

FIG S5 (A and B) AFM images of *P. denitrificans* cells in PBS incubated with anti-MVs antibodies ("anti-MVs" treatment). (C and D) Topographic and phase images of *P.*

denitrificans cells in PBS, incubated with (C) MVs plus anti-MVs antibodies ("MVs + anti-MVs" treatment) and with (D) anti-MVs antibodies ("anti-MVs" treatment). The AFM images were recorded at an imaging rate of 0.5 sec/frame and 100 × 100 pixels for panels A and B, and 2 sec/frame and 200 × 200 pixels for panels C and D.

Supplementary Movie Legends

Movie S1 Long-time AFM topological movie of the MV binding process to *P. denitrificans* cell surfaces. The movie took for 90 min after adding MVs into the AFM imaging chamber. AFM images were recorded at imaging rates of 2 sec/frame and 250 × 250 pixels.

Movie S2 Enlarged AFM topological movie trimmed from Movie S1 between 40:30–42:00 (min:sec). The white arrow indicates the MV particle that moves laterally on the cell surfaces.

Movie S3 Successive AFM phase imaging movie recorded during a phase shift alternation event on the cell surface.

Movie S4 3D AFM phase imaging of the "Decreasing MV" (left) and "Stable MV" (right). The AFM images were recorded at imaging rates of 2 sec/frame and 250 × 250 pixels.

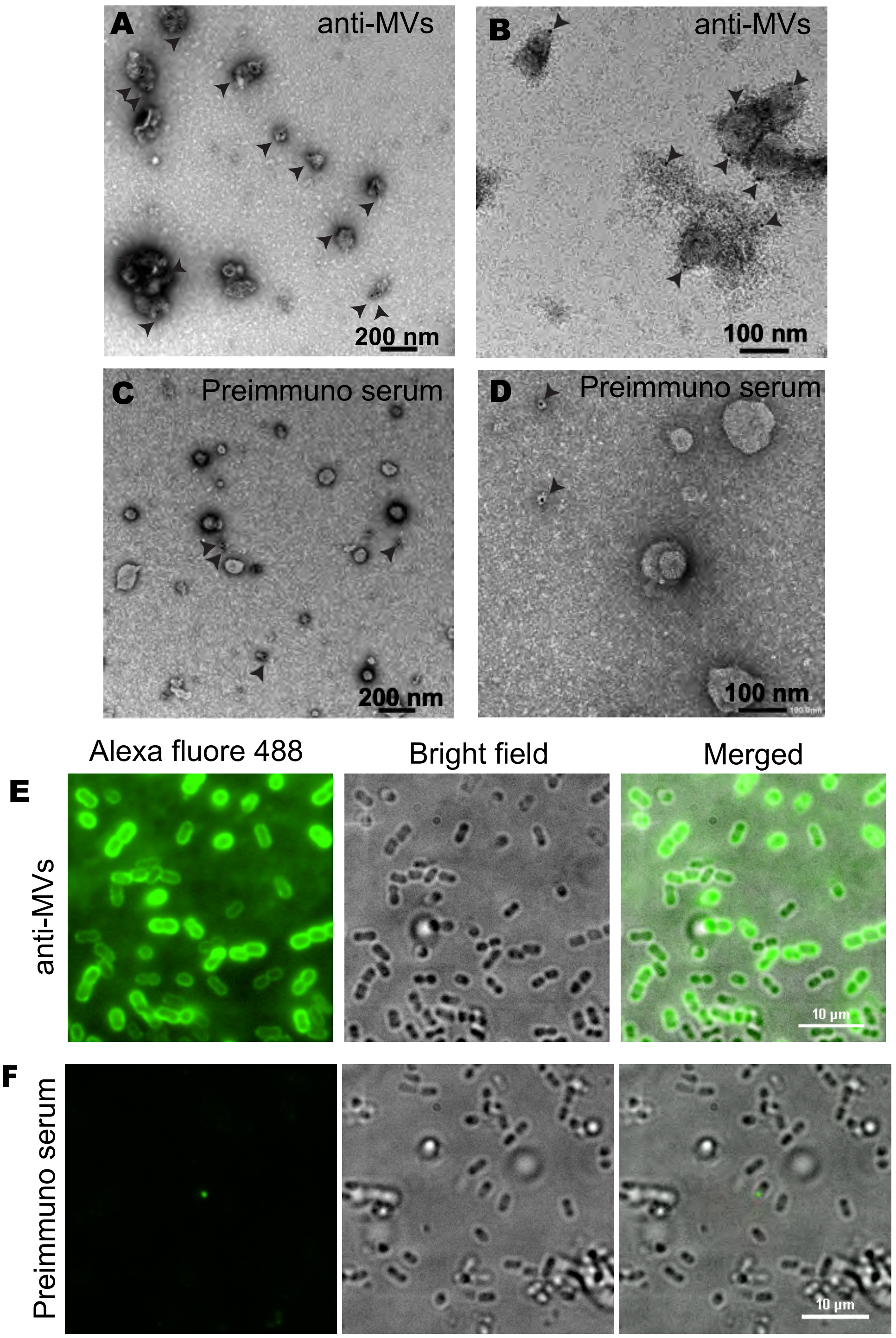

FIG S1 Transmission electron microscopic images of immunogold-stained *P. denitrificans* MVs with anti-MVs serum (A and B) and preimmune serum (C and D). Arrowheads indicate 15-nm (A and C) and 5-nm (B and D) gold particles. Each gold particle represents a complex of antigen, rabbit IgG, and anti-rabbit IgG antibody-gold conjugate. The gold particles specifically localized the MVs to anti-MVs antibodies (A and B). In contrast, significantly fewer gold particles could be observed at random positions in the negative control experiment using preimmune serum (C and D). (E and F) Immunofluorescence-staining of the AHL reporter cells labeled with (E) anti-MVs and (F) preimmune serum. Each fluorescence and bright-field image in panels E and F were acquired under the same microscopic conditions. Cell-bound IgGs were labeled with anti-rabbit IgG antibody Alexa Fluor 488 conjugates. The fluorescence signals were detected from the peripheral region of the cells, suggesting that the anti-MVs antibodies were bound to the cell surface. The immunogold-staining (A-D) and the immunofluorescence-staining (E and F) indicated that the anti-MVs antibodies recognize both the MV and *P. denitrificans* cell surfaces.

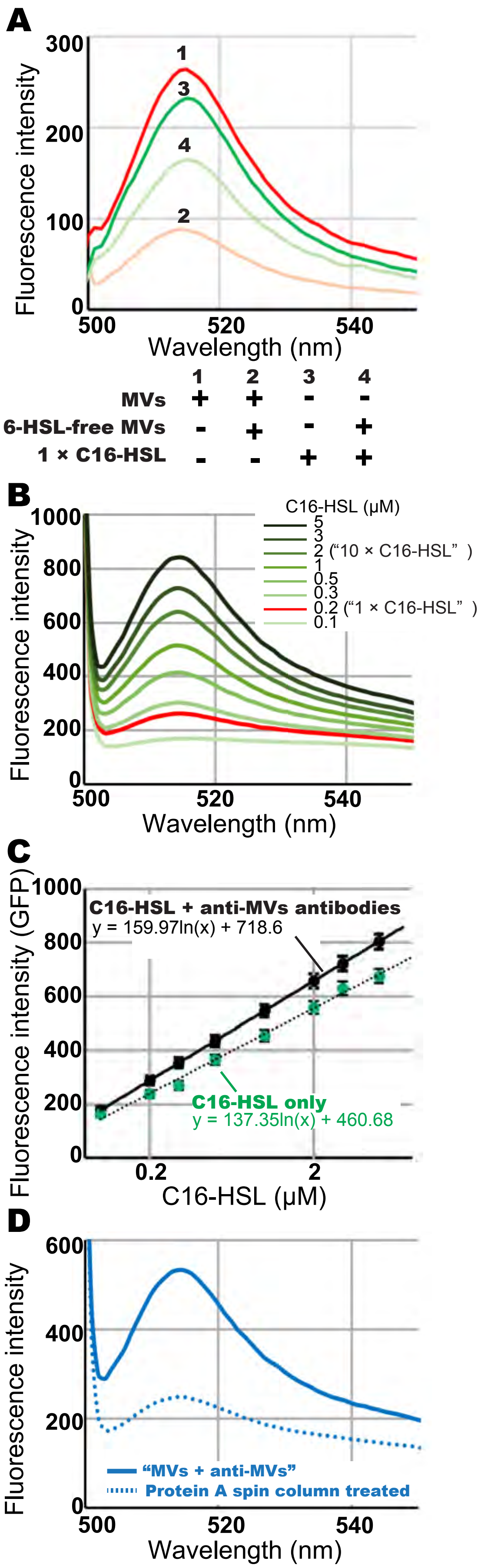

FIG S2 (A) Effects of C16-HSL-free MVs addition. Representative fluorescence spectra used as data for Fig. 1F. Fluorescence spectra of the AHL reporter cell suspensions incubated with MVs (1.1×10^9 particles/ml; “MVs” treatment, red), “MVs” treatment plus C16-HSL-free MVs (1.1×10^{10} particles/ml, light red), 0.2 μM C16-HSL (“1 × C16-HSL” treatment, green), and “C16-HSL” treatment plus C16-HSL-free MVs (1.1×10^{10} particles/ml, light green). (B) Fluorescence titration spectra of the AHL reporter cell suspensions incubated with purified anti-MVs antibodies-containing PBS (final concentration of 13 μg/ml) and C16-HSL ranging between 0.1–5 μM. (C) Relationship between C16-HSL concentrations and GFP fluorescence intensities in the presence of the anti-MVs antibodies estimated from fluorescence spectra in panel B (black). GFP fluorescence intensity values observed from emissions at 515 nm. The data are represented as the means ± SE (bars) (n = 3). The equation of the fitted curve is indicated. The green squares and the equation represent the relationship between C16-HSL concentrations and GFP fluorescence intensities without anti-MVs antibodies are highlighted in Fig. 1D for comparison. (D) Fluorescence spectra of the AHL reporter cell suspensions incubated with MVs plus anti-MVs antibodies (“MVs + anti-MVs” treatment, solid line) and the flow through of Protein-A Sepharose column (dashed line).

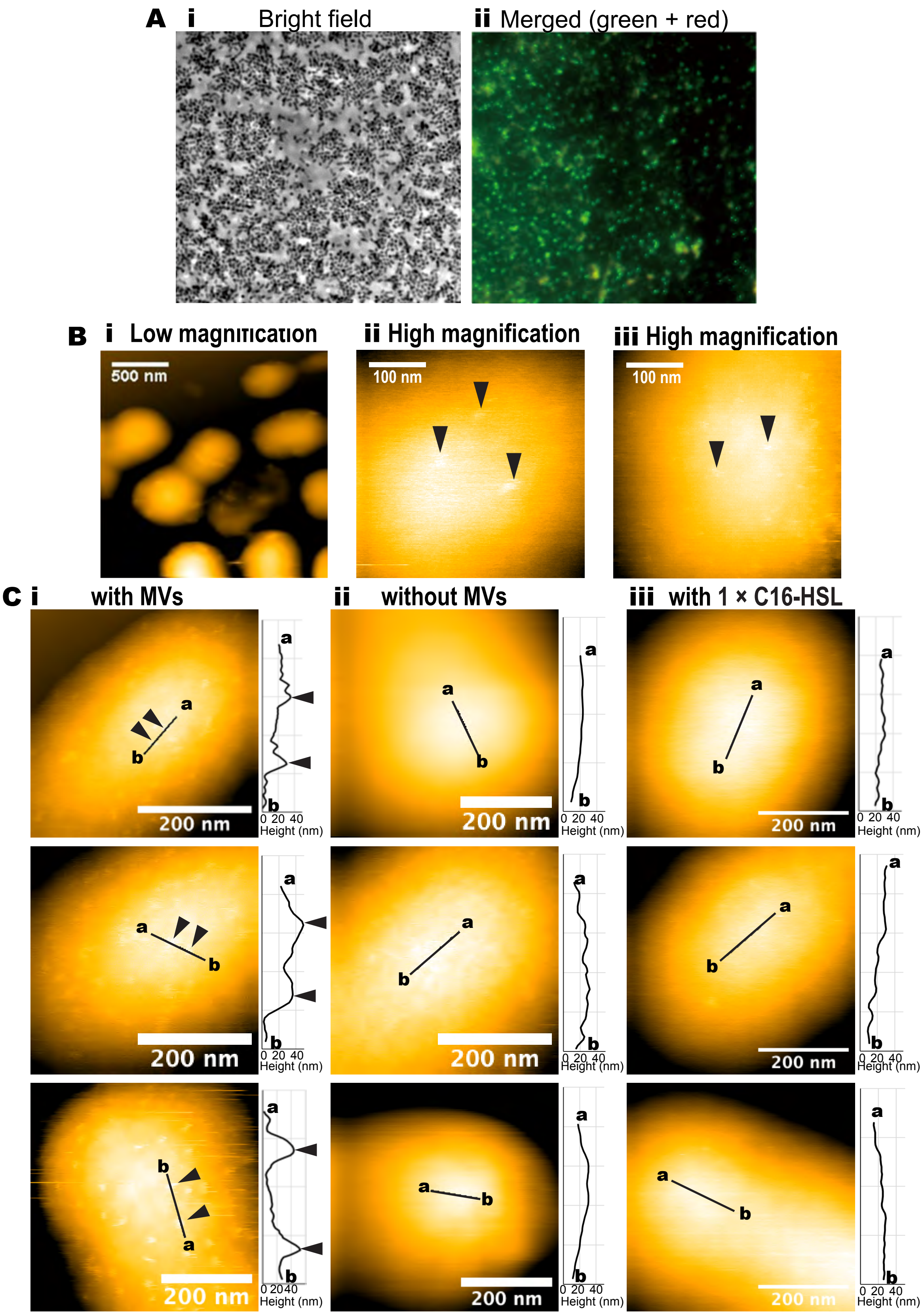

FIG S3 (A) Immobilized cell viability on a mica substrate assessed using the LIVE/DEAD BacLight bacterial viability kit. (Ai) Bright-field image of immobilized cells on a mica substrate. (Aii) Merged green and red fluorescence images of stained cells using the LIVE/DEAD BacLight bacterial viability kit. Almost all cells are stained in green fluorescence on the mica substrate. Live bacteria with intact membranes fluoresce in green, while those with damaged membranes in red. (B) AFM images of *P. denitrificans* cell surface in PBS. (Bi) Low-magnification images of *P. denitrificans cells*. (Bii and iii), High-magnification images of *P. denitrificans* cell surfaces. Arrowheads indicate globular small particles observed with non-treated cell surfaces. (C) Effect of the cell surface structure on the C16-HSL treatment. AFM images of *P. denitrificans* cell surface incubated with (Ci) MVs-containing PBS (1.1 × 10^9 particles/ml), (Cii) PBS, and (Ciii) 0.2 μM C16-HSL-containing PBS. The AFM imaging was performed 30 minutes after the incubations. The line profiles between a to b in each AFM image are indicated on the light sides. The AFM images were recorded at an imaging rate of 2.0 sec/frame and 200 × 200 pixels for low-magnification images in panel Bi, and at imaging rates of 0.5 sec/frame and 100 × 100 pixels for high-magnification images in panels Bii, Biii, and C.

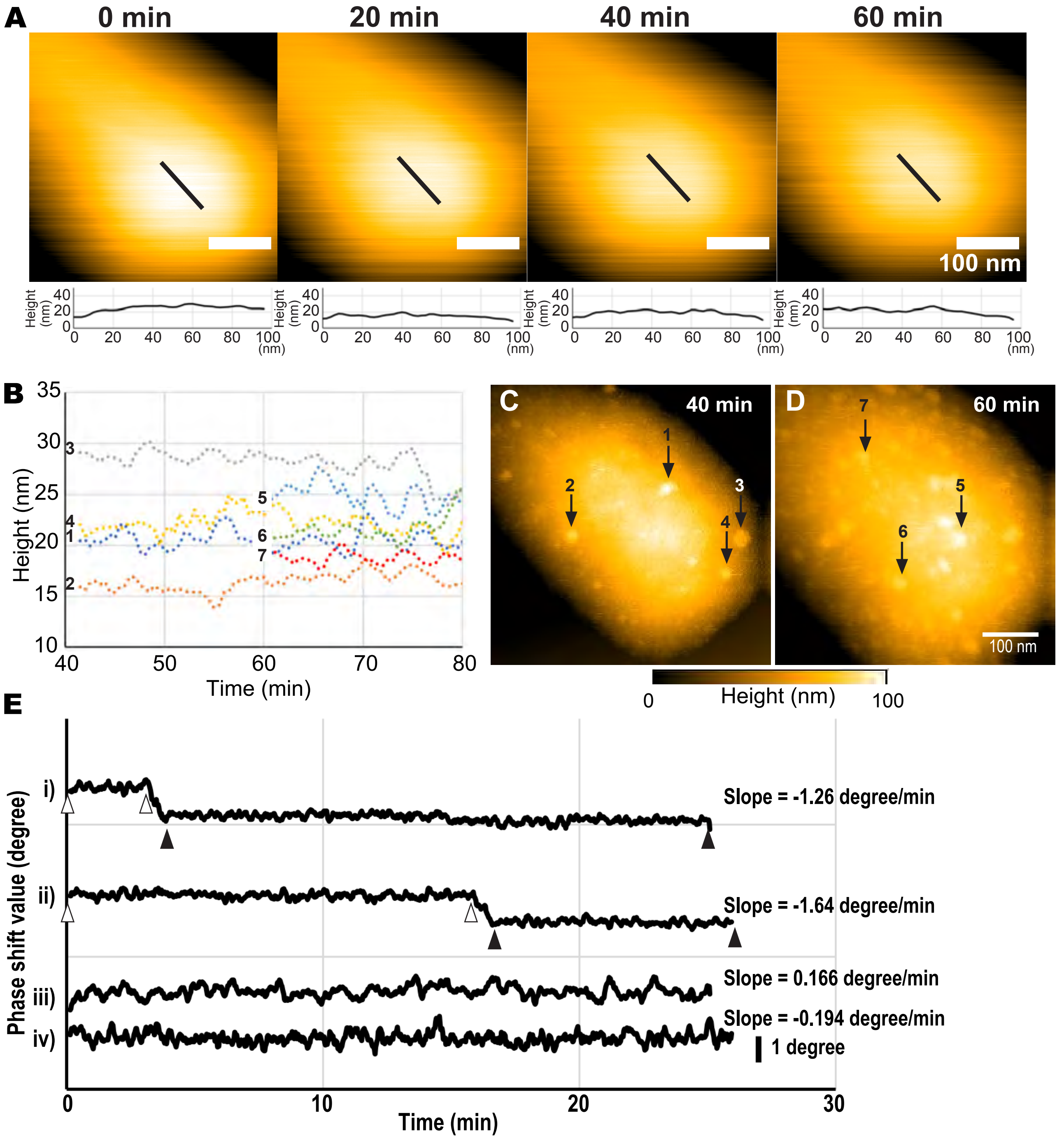

FIG S4 (A) Still images of the long-time successive AFM topological images of *P. denitrificans* cell surfaces in PBS without MVs. The AFM image was taken for 60 min at an imaging rate of 2.0 sec/frame. Long-time AFM imaging did not cause any apparent damage to the cell surface structure. The line profiles in each AFM image are highlighted at the bottom of each image. (B) Time-dependent structural alterations of MVs attached to the cell surface. The time courses of the height of seven MVs between 1–7. (C) Four MVs (1–4) were recorded from 40 min to 80 min, and (D) three MVs (5–7) were recorded from 60 min to 80 min after adding MVs (Movie S1). All heights of MVs on the cell surface were stable. We were unable to observe alteration of MV heights during the AFM observation on the cell surface. (E) The time courses of the representative MV phase shift values throughout the AFM observations. i), ii), and iii), iv) are time courses from “Decreasing” and “Stable” MVs, respectively. The maximum slope values in the time courses are highlighted. In i), and ii), the phase shift values before (between open triangles) and after (between solid triangles) the decreasing phase shift value were constant.

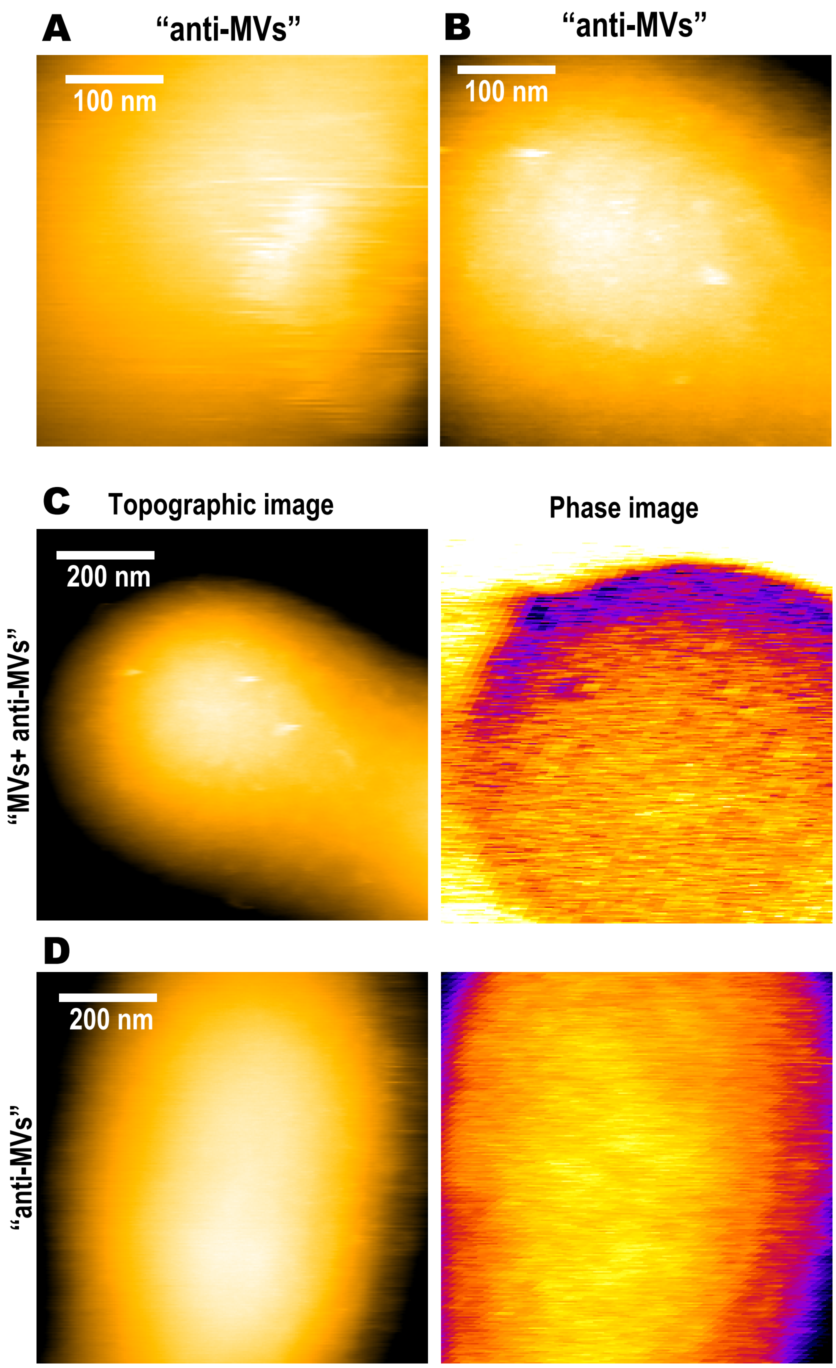

FIG S5 (A and B) AFM images of *P. denitrificans* cells in PBS incubated with anti-MVs antibodies ("anti-MVs" treatment). (C and D) Topographic and phase images of *P. denitrificans* cells in PBS, incubated with (C) MVs plus anti-MVs antibodies ("MVs + anti-MVs" treatment) and with (D) anti-MVs antibodies ("anti-MVs" treatment). The AFM images were recorded at an imaging rate of 0.5 sec/frame and 100 × 100 pixels for panels A and B, and 2 sec/frame and 200 × 200 pixels for panels C and D.
